# In Vitro Susceptibility of HIV Isolates with High Growth Capability to Antiretroviral Drugs

**DOI:** 10.3390/ijms232315380

**Published:** 2022-12-06

**Authors:** Alfredo A. Hinay, Kyosuke Kanai, Akeno Tsuneki-Tokunaga, Mizuki Komatsu, Elizabeth O. Telan, Seiji Kageyama

**Affiliations:** 1 Division of Virology, Department of Microbiology and Immunology, Faculty of Medicine, Tottori University, Yonago 683-8503, Japan; 2 National Reference Laboratory, STD AIDS Cooperative Central Laboratory, San Lazaro Hospital, Manila 1003, Metro Manila, Philippines

**Keywords:** human immunodeficiency virus, antiretroviral drugs, viral growth capability

## Abstract

It has been considered that reduced susceptibility to antiretroviral drugs is influenced by drug adherence, drug tolerance and drug-resistance-related mutations in the HIV genome. In the present study, we assessed the intrinsic high viral growth capability as a potential viral factor that may influence their susceptibility to antiretroviral drugs using an in vitro model. Phytohemagglutinin-activated peripheral blood mononuclear cells (1.5 × 10^6^ cells) were infected with HIV isolates (10^6^ copies/mL). The culture was carried out at different concentrations (0.001–20 μM) of 13 synthetic antiretroviral compounds (six nucleoside/nucleotide reverse transcriptase inhibitors, one non-nucleoside reverse transcriptase inhibitor, four integrase inhibitors, and two protease inhibitors), and HIV production was assessed using HIV-RNA copies in culture. The 90% inhibitory concentration (IC_90_) and pharmacokinetics of an antiretroviral agent were used as parameters to determine the reduced antiretroviral drug susceptibility of HIV isolates with high growth capability to synthetic antiretroviral compounds. The high growth capability of HIV isolates without any known drug resistance-related mutation affected their susceptibility to tenofovir (IC_90_ = 2.05 ± 0.40 μM), lamivudine (IC_90_ = 6.83 ± 3.96 μM), emtricitabine (IC_90_ = 0.68 ± 0.37 μM), and efavirenz (IC_90_ = 3.65 ± 0.77 μM). These antiretroviral drugs showed IC_90_ values close to or above the maximum plasma concentration against HIV isolates with high growth capability without any known drug resistance-related mutation. Our results may contribute to the development of effective strategies to tailor and individualize antiretroviral therapy in patients harboring HIV isolates with high growth capability.

## 1. Introduction

Despite the decrease in overall HIV-related mortality, recent data have shown that approximately 37.7 million people live with HIV/AIDS, which continues to be the underlying cause of mortality for about 1 million people annually [1]. Considering this challenge, sustained global efforts with the expansion of antiretroviral therapy have dramatically increased the number of people receiving HIV treatment in recent years, particularly in resource-limited countries. In 2020, 73% of all HIV-infected individuals received treatment, of which 66% were virally suppressed [2]. The primary goal of antiretroviral therapy is to suppress viral load to an undetectable level and prevent its transmission to other uninfected individuals [3,4,5].

Treatment options recommended by the World Health Organization (WHO) provide guidelines on when and what antiretroviral regimens should be administered to patients. According to the current WHO guidelines, the initial antiretroviral regimens used by most national treatment programs in resource-limited settings include the following three antiretroviral agents: one key antiretroviral drug, either a non-nucleotide reverse transcriptase inhibitor, integrase inhibitor, protease inhibitor; and two backbone antiretroviral drugs from among the nucleoside/nucleotide reverse transcriptase inhibitors [4]. These combined antiretroviral regimens provide excellent potency, safety, and tolerability, making lifelong viral suppression achievable [6].

However, the above-mentioned treatment options have been impaired frequently due to the reduced antiretroviral drug susceptibility [7]. This condition is typically associated with limited treatment monitoring, poor drug adherence and tolerance, and the emergence of resistance-related mutations during antiretroviral therapy [8,9,10,11,12]. Although the exact mechanism by which HIV viral factors compromise virological control remains unclear, it is likely to involve the intrinsic replication capability of the virus [11]. Recent studies showed that HIV replication capability appears to affect the response to antiretroviral therapy and is postulated to influence clinical outcomes [7,13,14,15]. Selhorst et al. demonstrated that a virus with high growth capability during early infection significantly contributed to disease progression, even during antiretroviral drug therapy [11]. Of note, our previous study showed various viral growth capabilities in vitro in clinical samples collected from the surveillance of drug-resistant strains in the Philippines and good relationships between growth capability and plasma viral load in vivo [16]. These results suggest that it may be challenging to suppress the replication of isolates with high growth capability.

In the present study, we examined the antiviral activity of synthetic antiretroviral compounds against HIV with high growth capability without any known drug resistance-related mutation in vitro.

## 2. Results

### 2.1. HIV Isolates Characteristics

**Figure 1:**
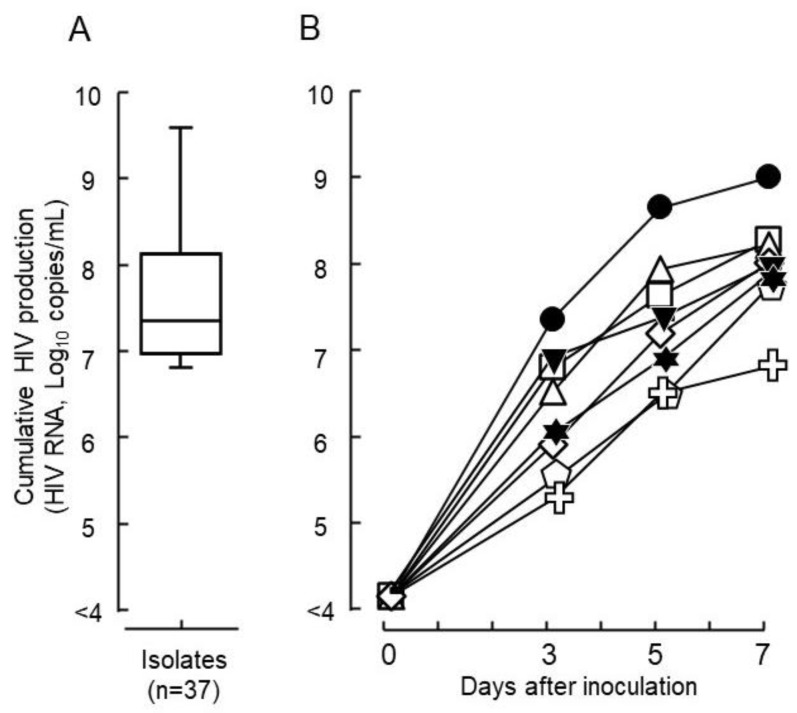
Growth capabilities of HIV epidemic isolates. (**A**) The wide distribution of growth capabilities was determined for the 37 clinical isolates tested and shown using production levels in culture with phytohemagglutinin-activated peripheral blood mononuclear cells for 7 days in vitro (mean ± standard error). (**B**) In vitro viral growth kinetics in the culture are shown for seven representative HIV isolates and one laboratory strain of HIV (● DR1509-397, ▼ DR1605-400, ⬠ S1506-064, ◻ DR1606-521, △ DR1510-726, ◇ DR1606-479, ✞ DR1606-559, and ✶ pNL4-3). Closed symbols denote drug sensitive isolates and open symbols denote drug-resistant isolates against tested nucleoside/nucleotide transcriptase inhibitors. Non-nucleoside/nucleotide reverse transcriptase inhibitors, and protease inhibitors based on the Stanford university HIV drug resistance database genotype-phenotype comparison scores.

From the 37 clinical samples collected during the surveillance of drug-resistant strains in the Philippines, diverse replication capabilities (5 × 10^6^–3 × 10^9^ copies/mL) were observed using a primary culture system of phytohemagglutinin-activated peripheral blood mononuclear cells (PHA-PBMCs) in vitro (Figure 1A). Seven representative isolates and one laboratory strain of HIVpNL4-3 were selected for the analysis of viral growth kinetics. Among these, six showed a higher virus yield at the end of the virus culture and one had a lower yield. Three different time points (days 3, 5, and 7) were evaluated in the culture to observe the viral growth kinetics using a primary culture system of PBMCs (Figure 1B, Table 1). After the inoculation of virus (10^6^ HIV-RNA copies), isolates DR1509-397, DR1606-521, DR1510-726, DR1605-400, DR1606-479 and S1506-064 multiplied to 2.0 × 10^9^, 3.4 × 10^8^, 3.0 × 10^8^, 2.3 × 10^8^, 1.8 × 10^8^, and 1.0 × 10^8^ copies/mL on day 7, respectively. HIVpNL4-3 produced a viral copy of 1.3 × 10^8^ copies/mL. In contrast, DR1606-559 showed limited growth, one-fold that of the inoculum size (9.7 × 10^6^ copies/mL) (Figure 1B, Table 1). Genotyping results showed that all isolates were CRF01_AE, except S1506-064, which was subtype B. Moreover, all isolates were CXCR4 viruses, except DR1606-559, which was CCR5, based on the coreceptor prediction (Table 1).

### 2.2. Screening for Antiretroviral Drugs

**Figure 2:**
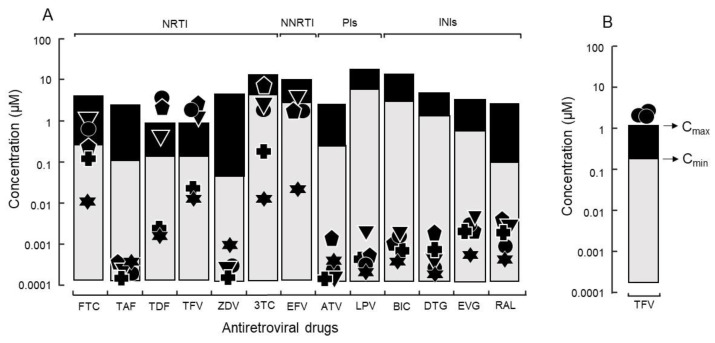
(**A**) The IC_90_ of antiretroviral drugs against isolates without known drug-resistance mutations and relations with C_min_ and C_max_. Thirteen synthetic antiretroviral compounds were evaluated: six nucleoside/nucleotide reverse transcriptase inhibitors (NRTIs) (FTC: emtricitabine, 3TC: lamivudine, TAF: tenofovir alafenamide, TFV: tenofovir, TDF: tenofovir disoproxil fumarate, and ZDV: zidovudine). One non-nucleoside reverse transcriptase inhibitor (NNRTI) (EFV: efavirenz); two protease inhibitors (PIs) (ATV: atazanavir and LPV: lopinavir); and four integrase inhibitors (INIs) (BIC: bictegravir, DTG: dolutegravir, EVG: elvitegravir, and RAL: raltegravir) against HIV with higher growth capability (● DR1509-397, ▼ DR1605-400, ⬟ S1506-064), lower growth capability (✚ DR1606-559), and laboratory strain of HIV (✶ pNL4-3). Because of the presence of drug-resistance mutations, isolates of ⬟ S1506-064 and ✚ DR1606-559 were not evaluated for ZDV and EFV, respectively. (**B**) Tenofovir response to isolate (● DR1509-397) with the highest growth capability, was evaluated using three different blood sources.

Representative HIV isolates with relatively high growth capabilities were subjected to drug screening using 13 synthetic antiretroviral compounds. None of the tested antiretroviral compounds exhibited 50% cytotoxicity at the maximum concentration used in the assay. Remarkably, the 90% inhibitory concentration (IC_90_) values were close to maximum plasma concentration (C_max_) and above minimum plasma concentration (C_min_) for some isolates with high growth capability and without any known drug resistance-related mutation. A typical case was the use of tenofovir against isolates with a high growth capability. Three isolates (DR1509-397, DR1605-400, and S1506-064) had IC_90_ values of 2.0 ± 0.40, 1.4 ± 0.87, and 2.8 ± 0.47 μM, respectively, which were higher than their C_max_ values (Figure 2A).

**Figure 3:**
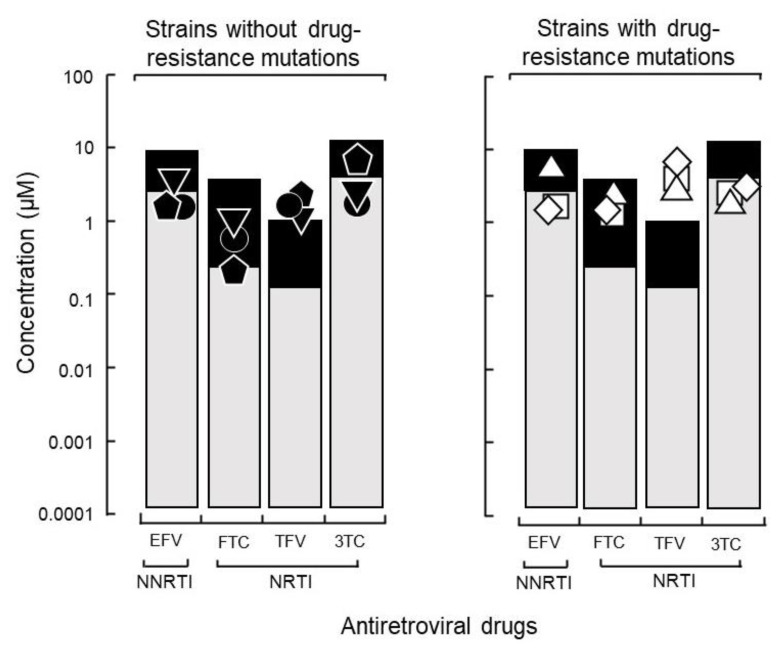
Influence of growth capability on the therapeutic effects of a TDF-based regimen. The 90% inhibitory concentrations (IC_90_) of three isolates with a high growth capability without drug resistance-related mutations (**left**: ● DR1509-397, ▼ DR1605-400, and ⬟ S1506-064) and three isolates with such mutations (**right**: ◻ DR1606-521, △ DR1510-726, and ◇ DR1606-479) were compared. Abbreviations are as in Figure 2.

**Figure 4:**
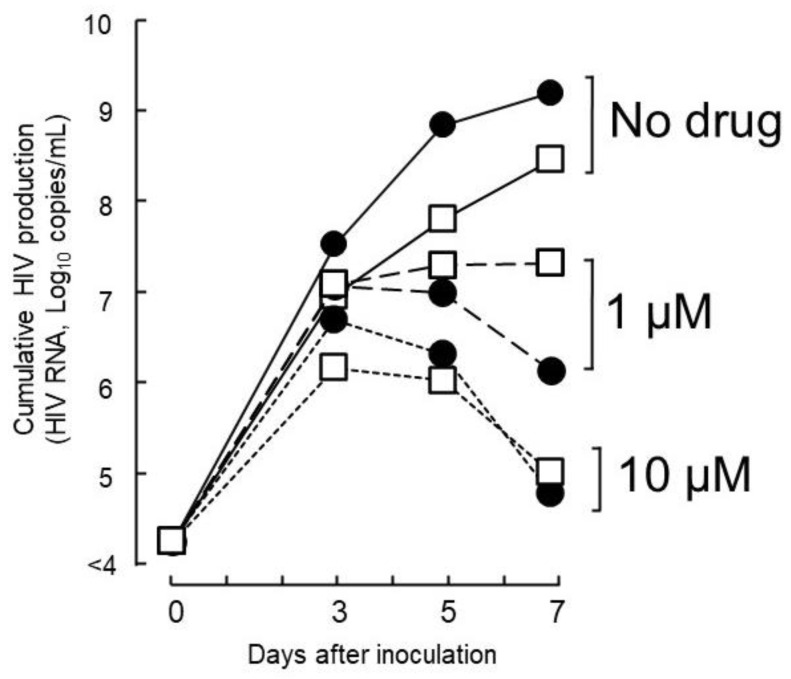
Anti-HIV activity of TFV against isolates without and with TDF-drug resistance-related mutations. The tested concentration of 1 µM corresponded to the C_max_ value of TFV, and 10 µM is the highest IC_90_ expected to significantly suppress the viral load of isolates without TDF drug resistance-related mutations (● DR1509-397) or with mutations (◻ DR1606-521). Abbreviations are as in Figure 2.

To determine whether host factors contributed to the viral load of isolates with high growth capabilities, DR1509-397 was screened using two additional blood sources, even in the presence of tenofovir. The results were still consistent with a mean IC_90_ of 1.9 ± 0.04 μM (Figure 2B). As expected, isolates with drug resistance-related mutation (DR1606-521, DR1510-726, and DR1606-479) had higher IC_90_ values for tenofovir (8.3 ± 0.12, 7.7 ± 0.06, and 8.5 ± 0.10 μM), respectively, which were above the C_max_ (Figure 3). Moreover, the in vitro growth behavior of the tenofovir-sensitive (DR1509-397) and tenofovir-resistant (DR1606-521) isolates showed the same pattern in the presence of tenofovir at 1 μM (close to C_max_). Surprisingly, the isolate with high growth capability hardly showed the decrease in virus production, similar to that of the isolate with drug resistance-related mutations. An unreachable concentration in vivo (IC_90_: 10 μM, above C_max_) was required to fully suppress the number of viral copies in both isolates (Figure 4).

**Figure 5:**
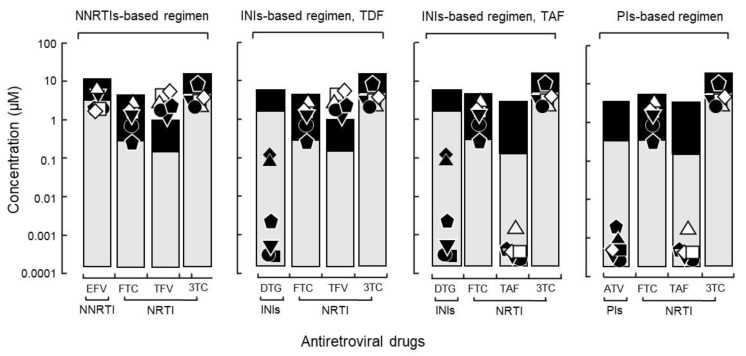
Application of the IC_90_ and its relationship with C_min_/C_max_ to WHO recommended regimens. The levels of IC_90_, C_min_ and C_max_ are shown for an NNRTI-, INI-based regimen with DTG and TFV (active form of TDF in plasma), INI-based regimen (alternative) with TAF, and PI-based regimens including ATV (● DR1509-397, ▼ DR1605-400, and ⬟ S1506-064, ◻ DR1606-521, △ DR1510-726, ◇ DR1606-479). Closed symbols were assigned to all isolates in DTG because integrase resistance mutations were not assessed. Abbreviations are as in Figure 2.

An non-nucleoside/nucleotide reverse transcriptase inhibitor regimen containing efavirenz, which is used by most national treatment programs in resource-limited settings, showed IC_90_ values above C_min_ both with and without any known drug resistance-related mutation (Figure 5). Efavirenz failure could be replaced by the integrase inhibitor dolutegravir and protease inhibitor atazanavir, which remarkably suppressed the viral yield of HIV isolates in vitro (Figure 5). Among the 13 antiretroviral synthetic compounds, atazanavir, tenofovir alafenamide, zidovudine, and dolutegravir showed the lowest IC_90_ values of 1.30 ± 1.12, 0.30 ± 0.23, 1.50 ± 1.11, and 1.10 ± 0.72 nM, respectively. All drugs were highly active against HIVpNL4-3.

## 3. Discussion

In vitro viral growth kinetics in PHA-PBMC cultures was demonstrated for isolates with diverse growth capabilities. HIV isolates with higher growth capability but without any known drug resistance-related mutation were screened for antiretroviral drugs, and eight compounds (atazanavir, bictegravir, dolutegravir, elvitegravir, lopinavir, raltegravir, tenofovir alafenamide, and zidovudine) retained susceptibility with lower IC_90_ values. However, lamivudine, emtricitabine, and efavirenz had IC_90_ values that were close to or above C_min_. A compound of tenofovir, the active form of tenofovir disoproxil fumarate in the plasma and the backbone drug in the first-line antiretroviral regimen, showed IC_90_ values above C_max_.

The extent to which HIV replicates during antiretroviral therapy remains controversial [17] and most studies have associated drug resistance with drug resistance-related mutations [12,18,19]. However, as described above, viral copies of HIV isolates can still be high, even when they are not affected by the resistance-related mutations in efavirenz, emtricitabine, tenofovir, or lamivudine. The computed IC_90_ values were similar to those of isolates with drug resistance-related mutations. The difficulty that efavirenz, emtricitabine, tenofovir, and lamivudine found in inhibiting HIV production in PHA-PBMC cultures stands in conflict with a large body of evidence that suggests they are effective in suppressing HIV replication [4,20,21,22,23]. Our results suggest that HIV gains a replicative advantage and produces increased viral copies with drugs such as nucleoside/nucleotide reverse transcriptase inhibitors and non-nucleotide reverse transcriptase inhibitors because of its intrinsic viral growth capability. The reduced antiretroviral susceptibility of HIV isolates with a high growth capability to these antiretroviral drugs is critical because these regimens are the most popular first-line HIV treatments worldwide and are still included in the 2021-updated WHO recommendations [24].

The attained IC_90_ of tenofovir compared to that of tenofovir alafenamide presented in this study showed a potential advantage of tenofovir alafenamide in suppressing HIV isolates with high growth capability. Tenofovir alafenamide has also been found to be active against HIV isolates with drug resistance-related mutations in tenofovir disoproxil fumarate. Ex vivo clinical studies have also indicated that tenofovir alafenamide achieves a high intracellular concentration of the active drug tenofovir diphosphate [25,26] and that most viruses harboring major nucleoside/nucleotide reverse transcriptase inhibitor drug resistance-related mutations are fully suppressed but not inhibited by tenofovir, although tenofovir prodrugs (tenofovir disoproxil fumarate and tenofovir alafenamide) have similar resistance profiles [27,28]. Similar to that observed ex vivo [23,29], the results of this study demonstrated that tenofovir alafenamide maintains its antiviral activity and can be used if tenofovir disoproxil fumarate failure is observed. Our results also provide evidence that most of the antiretrovirals tested, such as tenofovir alafenamide, zidovudine, protease inhibitors, and integrase inhibitors, were highly effective in suppressing viral copies in vitro. Moreover, integrase inhibitors have been shown to exhibit potent in vitro antiviral activity in multiple cell-based assays [4,30,31] and in vivo efficacy in clinical studies [32,33,34].

As described above, the reduced susceptibility of high growth capability isolates to antiretroviral drugs emphasizes the importance of considering the high growth capability of epidemic strains. Further analyses are needed to identify the genes responsible for growth capability and invent a high throughput system of screening assay. Moreover, prospective studies are required to evaluate the clinical significance of HIV isolates with high growth capabilities and the subsequent virological response to antiretroviral therapy.

## 4. Materials and Methods

### 4.1. HIV Isolates

From the 37 HIV strains previously isolated as part of the surveillance program on drug-resistant isolates in the Philippines [16], seven representative HIV isolates and one laboratory strain of HIVpNL4-3 (originated from HIV-1 infectious molecular clone, pNL4-3, provided by the NIH AIDS Reagent Program) were used in the study. Among these, HIV isolates DR1509-397, DR1605-400, S1506-064 (except zidovudine), and DR1606-559 (except efavirenz) did not have any known drug resistance-related mutation against the antiretroviral drugs used in the study. HIV isolates DR1606-479, DR1606-521, and DR1510-726 have drug resistance-related mutations in nucleoside/nucleotide reverse transcriptase inhibitors/non-nucleotide reverse transcriptase inhibitors and protease inhibitors according to the HIV Drug Resistance Database at Stanford University (http://hivdb.stanford.edu/, accessed on 26 April 2022). Aliquots of HIV isolates were prepared and stored in a deep freezer (−75 °C) until ready for use.

### 4.2. Growth Capability Assessment

HIV-seronegative PBMCs were stimulated with phytohemagglutinin-P (PHA-P; 2.0 µg/mL) in RPMI1640 complete medium supplemented with recombinant interleukin 2 (IL-2, 10 ng/mL; Genzyme, Cambridge, MA, USA) and 10% fetal bovine serum for 48–72 h to promote T cell blast formation. PHA-PBMCs (1.5 × 10^6^ cells) were inoculated with 100 μL of HIV epidemic isolates (10^6^ HIV-RNA copies/mL) and incubated at 37 °C for 1 h. To remove unbound and non-infectious viruses, the cells were washed twice with RPMI1640 medium and resuspended in complete medium to a final volume of 1 mL in 24-well plates. Half of the culture medium was replaced on day 3 to maintain the culture conditions of PBMCs. The culture supernatants on days 3, 5, and 7 were subjected to RNA extraction and quantitative one-step RT-PCR to create growth curves for the representative HIV isolates.

### 4.3. Drug-Induced Cytotoxicity Assay

HIV-seronegative PBMCs were incubated with various concentrations of antiretroviral compounds in 96-well plates for 72 h. Cell toxicity was then evaluated using the Cell Counting Kit-8 (CCK-8) (DOJINDO Laboratories, Kumamoto, Japan) [35]. The absorbance was measured at 450 nm using a 96 well plate reader (AS ONE MPR-A100; AS ONE Corporation, Tokyo, Japan).

### 4.4. Evaluation of Drug Response

The activity of 13 synthetic antiretroviral compounds at concentrations ranging from 0.001 to 20 μM was evaluated against HIV isolates with diverse growth capabilities and without any known drug resistance-related mutation. The following nucleotide reverse transcriptase inhibitors were used: emtricitabine (Tokyo Chemical Industry, Tokyo, Japan), lamivudine (Tokyo Chemical Industry, Japan), tenofovir (Selleck Biotech, Tokyo Japan), tenofovir alafenamide (Selleck Biotech, Japan), tenofovir disoproxil fumarate (Tokyo Chemical Industry, Japan), and zidovudine (FUJIFILM Wako, Tokyo, Japan). As it was reported that tenofovir and its prodrugs (tenofovir disoproxil fumarate and tenofovir alafenamide) are measurable in plasma, the antiviral activity of these three synthetic drugs were assessed in vitro [25,36]. In addition, one non-nucleotide reverse transcriptase inhibitor, efavirenz (Tokyo Chemical Industry, Japan); two protease inhibitors, atazanavir (Selleck Biotech, Japan) and lopinavir (Funakoshi Frontiers in Life Science, Tokyo, Japan); and four integrase inhibitors, bictegravir (Selleck Biotech, Japan), dolutegravir (Selleck Biotech, Japan), elvitegravir (EVG, Selleck Biotech, Japan), and raltegravir (Selleck Biotech, Japan) were analyzed. Representative HIV isolates and a laboratory strain of HIVpNL4-3 were screened for antiretroviral drugs. Briefly, PHA-PBMCs (1.5 × 10^6^ cells) were pelleted at 1000 g for 5 min, resuspended with an HIV preparation (10^6^ copies) in 100 µL volume, and incubated at 37 °C for 1 h. To remove unbound and non-infectious viruses, the cells were washed twice with RPMI1640 medium and then cultured in different concentrations of antiretroviral compounds (1 mL). The culture was carried out for 7 days, as described above, for growth capability assessment. All procedures were performed in triplicate.

### 4.5. Evaluation of Antiretroviral Susceptibility

To determine the susceptibility of HIV isolates with high growth capability to synthetic antiretroviral compounds, we used the in vitro susceptibility of the virus (IC_90_) and pharmacokinetics of an antiretroviral agent (compared to reported C_min_ and C_max_). The reduced antiretroviral drug susceptibility of HIV isolates was reflected in an increase in the inhibitory concentration to a certain level close to or higher than the C_min_–C_max_ range [37].

### 4.6. RNA Extraction and Polymerase Chain Reaction

HIV-RNA in the culture medium (140 µL) was extracted using a QIAamp Viral RNA Mini Kit (QIAGEN KK-Japan, Tokyo, Japan) and subjected to quantitative reverse transcription polymerase chain reaction (qRT-PCR). The amount of HIV-RNA was assayed using a Luna Universal One-Step qRT–PCR kit (New England Biolabs Japan, Tokyo, Japan) and forward (GagB-1F, 5′-AGTGGGGGGACATCAAGCAGCCATGCAAAT-3′, HXB2 position: 1359–1388) and reverse (GagB-1R, 5′-TGCTATGTCACTTCCCCTTGGTTCTCT-3′, 1500–1474) primers. The PCR signal was assessed by comparison with a standard curve (encompassing 10^2^–10^8^ copies/reaction of the in vitro-transcribed RNA transcripts).

### 4.7. HIV Pol Region Nucleotide Sequences and Genotyping

HIV-RNA in the pol region (HXB2:1827–3528) was reverse-transcribed and amplified in the first round of PCR with primers F1849/R3500 [16,38] using the SuperScript III One-Step Real-Time RT-PCR System with Platinum Taq DNA Polymerase (Thermo Fisher Scientific, Tokyo, Japan). Two HIV pol regions were amplified in the second round of PCR with DRPRO5/DRPRO2L [16,38] and DRRT1L/DRRT4L [16,38] using AmpliTaq Gold 360 Master Mix (Thermo Fisher Scientific, Waltham, MA, USA). The annealing temperature and elongation time were 57 °C and 90 s for the first PCR, and 55 °C and 60 s for the second PCR, respectively [16]. Subtype assignments were compared to those from Stanford-v8.1, COMET (COntext-based Modeling for Expeditious Typing), Luxembourg Institute of Health, and REGA HIV Subtyping Tool—Version 3.0—Stanford University [39]. The sequences described in this study have been deposited in the International Nucleotide Sequence Database under the accession numbers ON229916-ON229922 (Table 1).

### 4.8. HIV V3 Region Nucleotide Sequences and Coreceptor Prediction

The HIV env region was reverse-transcribed and amplified in the first round of PCR with the forward primer SQV3F1 [40] and reverse primer CO602 [40] using a One *Taq* One-Step RT-PCR kit (New England Biolabs). The HIV V3 region was amplified in the second round of PCR with the forward primer SQV3F2 [40] and reverse primer CD4R [40] using AmpliTaq Gold 360 Master Mix (Thermo Fisher Scientific, Waltham, MA, USA). The annealing temperature and elongation time were 57 °C and 30 s for the first PCR and 55 °C and 60 s for the second PCR, respectively. Coreceptor usage prediction from genotype was employed using geno2pheno (version 2.5), and sequences were confirmed using the HIV sequence locator tool [41].

### 4.9. Data Analysis

The IC_50_ was calculated using a nonlinear regression curve fit of the dose response curves of each transformed drug concentration and normalized viral load (copies/mL) using GraphPad Prism software (version 8.0). The IC_90_ was determine using the IC_50_ and Hill slope factor from the curve fit in the online GraphPad calculator (https://www.graphpad.com/quickcalcs/ECanything1/, accessed on 1 October 2022) for effective concentration [42]. The results are expressed as the mean IC_90_ of three values obtained in triplicate for each isolate.

## 5. Conclusions

Reduced antiretroviral drug susceptibility was observed with tenofovir, lamivudine, emtricitabine, and efavirenz, even in the absence of specific drug resistance-related mutations in HIV isolates with high growth capability. This evidence, if not all, as a limited number of high growth capability isolates were tested against antiretroviral therapy, suggests the need for surveillance to strengthen the concept that the intrinsic viral growth capability is attributable to viral isolates and may influence their susceptibility to antiretroviral drugs. Moreover, binding thermodynamic analysis could provide additional information about the behavior of antiretroviral drugs and their response to the intrinsic high viral growth capability of HIV. However, that remains to be carried out in the further study. Furthermore, the development of high-throughput assays should be applied for efficient and less time-consuming assays. Finally, this study may aid in the development of diagnostic tools to identify this viral factor as an additional determinant of treatment failure and allow antiretroviral therapy to be tailored appropriately.

## Figures and Tables

**Table 1:** Characteristics of seven representative strains tested for drug response.

Strain ID	Amount of HIV-RNA (×10^6^ Copies/mL) in		Drug Resistance-Related Mutation ^c^	Genotype	Coreceptor Usage ^d^	Accession Number
	Culture Sup ^a^	Plasma ^b^				
DR1509-397	2000	1.6	None	CRF01_AE	CXCR4	ON229916
DR1606-521	340	1.7	EFV, FTC, TDF, 3TC	CRF01_AE	CXCR4	ON229919
DR1510-726	300	7.8	EFV, FTC, TDF, 3TC	CRF01_AE	CXCR4	ON229920
DR1605-400	230	0.74	None	CRF01_AE	CXCR4	ON229917
DR1606-479	180	5.1	EFV, FTC, TDF, 3TC	CRF01_AE	CXCR4	ON229921
S1506-064	100	Not done	ZDV	Subtype B	CXCR4	ON229918
DR1606-559	9.7	5.6	EFV	CRF01_AE	CCR5	ON229922

^a^ Maximum amount of HIV-RNA detected in culture supernatant of PHA-PBMC. ^b^ Patient’s plasma viral load. ^c^ Interpreted using Stanford university HIV drug resistance database. ^d^ Interpreted using Geno2pheno [coreceptor] 2.5, Max Planck Institute for Informatics. EFV: Efavirenz, FTC: emtricitabine, TDF: tenofovir disoproxil fumarate, 3TC: lamivudine, ZDV: zidovudine.

## Data Availability

All data generated or analyzed during this study are included in this published article.
